# Wound Healing Effects of *Aloe muth-muth*: In Vitro Investigations Using Immortalized Human Keratinocytes (HaCaT)

**DOI:** 10.3390/biology9110350

**Published:** 2020-10-23

**Authors:** Morné Fouché, Clarissa Willers, Sias Hamman, Christiaan Malherbe, Jan Steenekamp

**Affiliations:** 1 Centre of Excellence for Pharmaceutical Sciences, North-West University, Private Bag X6001, Potchefstroom 2520, South Africa; 24109207@student.g.nwu.ac.za (M.F.); sias.hamman@nwu.ac.za (S.H.); jan.steenekamp@nwu.ac.za (J.S.); 2 Plant Bioactives Group, Post-Harvest and Agro-Processing Technologies, Agricultural Research Council (ARC), Infruitec-Nietvoorbij, Stellenbosch 7599, South Africa; malherbech@arc.agric.za

**Keywords:** *Aloe muth-muth*, cell migration, HaCaT cells, in vitro cytotoxicity, scratch assay, wound healing

## Abstract

**Simple Summary:**

*Aloe muth-muth* is a cross-bred species cultivated from the well-known Aloe plants, namely *Aloe vera* and *Aloe ferox* through forced pollination. Considering that Aloe plants were traditionally widely used for treatment of wounds and skin lesions, *Aloe muth-muth* was also thought to have possible wound healing properties. Therefore, this study tested the ability of parts from the *Aloe muth-muth* plant to improve the closure of wounds induced in human skin cell layers grown in an incubator in a laboratory. Both the whole leave material and the inner gel-like part of this plant were tested for wound healing properties. It was found that the *Aloe muth-muth* gel part taken from the leaves possesses very high wound healing activity and can possibly be used in future wound treatments.

**Abstract:**

The traditional use of *Aloe* spp. for the purpose of wound healing has a long history and is widespread internationally. Recently, a hybrid aloe plant (*Aloe muth-muth*) has been cultivated by cross pollination between *Aloe vera* and *Aloe ferox*. The *Aloe muth-muth* plant has not yet been investigated for medicinal properties and provides an opportunity for potential biological activity, including wound healing. The aim of this study was to investigate the in vitro wound healing effects of both *Aloe muth-muth* gel and whole leaf material with the use of the immortalized human keratinocyte (HaCaT) cell line. Cell viability was conducted using methyl thiazolyl tetrazolium (MTT) assays. In vitro wound healing was tested on HaCaT cells using an established scratch assay method. The effect of *Aloe muth-muth* gel material on HaCaT cell migration was also investigated. *Aloe muth-muth* gel material exhibited statistically significantly (*p* < 0.05) higher percentage wound closure compared to the control at all three concentrations investigated. These findings confirm that this newly cultivated species, *Aloe muth-muth*, also possesses wound healing activity corresponding to that reported for the two species it is derived from, namely, *Aloe vera* and *Aloe ferox*. Therefore, *Aloe muth-muth* has the potential to be used in future wound therapeutics.

## 1. Introduction

A wide variety of products, substances, and dosage forms, such as alginates, antimicrobials, foams, and hydrocolloids, amongst others, are used in the treatment of wounds [1]. Phytochemicals that are found in traditional wound healing remedies commonly have antioxidant or anti-inflammatory properties [2,3]. Plants of the *Terminalia* genus (e.g., turmeric) are examples of such remedies used in Asian traditional wound healing remedies [2,4]. Other plant species that have been investigated for wound healing include *Anagallis arvensis* L. and *Anagallis foemina* Mill., for which anti-inflammatory and bacteriostatic properties (in vitro) have been reported [5]. Similarly, *Bulbine frutescens* and *Bulbine natalensis* have been found to exhibit significant improvement in wound contraction compared to an untreated control in animal studies [6]. The use of *Aloe* plant material as a wound healing remedy is especially notable and has been described, by Dioscorides, as early as the first century A.D. [7,8]. The wound healing effects of materials from different *Aloe* species have been investigated scientifically, both in vitro and in vivo. Increased wound area reduction was reported in mice treated with *Aloe vera* powder, in combination with poly(lactic-co-glycolic acid) (PLGA) nanofiber dressings and recombinant human epidermal growth factor (rhEFG), in comparison with controls of PLGA and rhEGF only [9]. *Aloe vera, Aloe ferox*, and *Aloe marlothii* leaf materials (gel and whole leaf extracts) have demonstrated increased wound healing in a zone exclusion-type assay using immortalized human keratinocytes (HaCaT cells) [10]. The in vitro wound healing potential of *Aloe vera* has also been demonstrated using human primary epidermal keratinocytes (HPEK) and a human skin equivalent model, with increased expression of integrin receptors (β1, α6, and β4) and E-cadherin being observed in HPEK treated with *Aloe vera* [11]. *Aloe muth-muth* has recently been cultivated by means of forced cross-pollination between *Aloe vera* and *Aloe ferox* at Rooiklip nursery in Swellendam, South Africa. It has thorny leaves that resemble those of *Aloe vera*, but also features erect racemes of yellow flowers. However, the medicinal properties such as the wound healing potential of *Aloe muth-muth* is still unknown.

Consequently, this study aimed to determine the cytotoxicity, wound healing, and migratory activity of *Aloe muth-muth* gel and whole leaf material using the human keratinocyte (HaCaT) cell line.

## 2. Materials and Methods

### 2.1. Preparation of Aloe Muth-Muth Whole Leaf and Gel Materials

Leaves of *Aloe muth-muth* plants were provided by Mr. Jaap and Hannes Viljoen of Rooiklip Nursery in Swellendam, South Africa. A voucher was deposited at the North-West University herbarium with accession number PUC0014886. The inner gel material and outer rind were separated by manually filleting the leaves with knives [10]. This filleting process basically entails cutting off the leaf base and tapering point, then peeling off the outer rind from the top and bottom sides, leaving an inner gel-like fillet. After rinsing with water, the inner gel material was liquidized in a kitchen blender and the outer rind parts were pulverized using a Retsch MM400 mixer mill (Retsch GmbH, Haan, Germany). To prepare *Aloe muth-muth* whole-leaf material, we added a quantity of the rind to the gel material in a 1:1 ratio that reflects the approximate ratio of gel to rind in a real *Aloe* leaf. Separate powdered materials were obtained by freezing the gel material and the whole leaf at −80 °C, followed by lyophilization with a VirTis freeze-dryer (Winchester, United Kingdom) until completely dry.

### 2.2. Characterization of Aloe Muth-Muth Whole Leaf and Gel Materials

The *Aloe muth-muth* gel and whole leaf plant materials were chemically characterized with quantitative proton nuclear magnetic resonance spectroscopy (^1^H-NMR), and spectra were obtained with a Bruker Avance III HD NMR (Bruker Corporation, Billerica, MA, USA). The quantities of marker molecules (i.e., aloverose, glucose, malic acid, lactic acid, citric acid, and whole leaf marker) in the *Aloe muth-muth* gel and whole leaf materials were determined according to a previously published method [12].

### 2.3. Culturing of HaCaT Cells

HaCaT cells were cultured in high glucose Dulbecco’s modified Eagle’s medium (DMEM; HyClone, Separations, Johannesburg, South Africa), supplemented with 10% fetal bovine serum (FBS) (ThermoFisher Scientific, Johannesburg, South Africa), 1% penicillin/streptomycin (10,000 U/mL, Lonza, Whitehead Scientific (Pty) Ltd., Cape Town, South Africa), 1% non-essential amino acids (NEAA) (Lonza), and 2 mM L-glutamine (Lonza). The cells were maintained in a humidified atmosphere at 37 °C and 5% CO_2_ using an ESCO CelCulture CO_2_ incubator (ESCO Technologies Inc., Horsham, PA, USA). Cells were cultured in T75 cm^2^ flasks. Growth medium was changed every 48 to 72 h and cells were viewed under a Nikon TS100 light microscope (Nikon Instruments, Tokyo, Japan) in order to estimate confluence.

### 2.4. Sub-Culturing of HaCaT Cells

HaCaT cells were sub-cultured by trypsinization at 70% to 80% confluency. Spent growth medium was removed from the culture flasks and the cells were rinsed twice with 10 mL phosphate-buffered saline (PBS; HyClone) to remove any residual medium. After we added 3 mL trypsin-ethylenediaminetetraacetic acid (EDTA) (Lonza), the flasks were incubated for 9-12 min at 37 °C. To neutralize the trypsin, we added 6 mL preheated growth medium to the flasks and washed the mixture thoroughly to remove all the cells from the flask surface. The cell suspensions were centrifuged at 140× *g* for 5 min and the supernatant was removed without disturbing the cell pellets. The pellets were then resuspended in growth medium and the suspension was divided using a 1:15 to 1:20 ratio into new flasks. Preheated growth medium was then added to the flasks to a final volume of 15 mL and the flasks returned to the incubator.

### 2.5. Methyl Thiazolyl Tetrazolium (MTT) Cell Viability Assay

Viability was measured after 24 and 48 h with the MTT assay [13] in order to evaluate the cytotoxicity of the plant materials at various concentrations. The experimental groups consisted of three concentrations of *Aloe muth-muth* gel or whole leaf (0.4, 0.6, and 1.3 mg/mL) [10]. In addition to the experimental groups, we included a dead cell control (treated with Triton X-100) and an untreated control, as well as a dimethyl sulfoxide (DMSO) blank group of wells. All experimental groups were tested in triplicate, whereas the control groups were tested in six replicates.

Cells were cultured to 80% confluence, trypsinized, and had their viable cell count determined using trypan blue exclusion. Cells were seeded in 96-well plates at 125,000 cells/mL (200 µL/well) and incubated for 24 h. After the 24 h incubation, the medium of the experimental groups were removed and replaced with medium containing plant material added at the relevant experimental concentrations. The experimental concentrations were obtained by preparing stock solutions with supplemented DMEM and then further diluting the stock solution to the relevant experimental concentration. Prior to dilution, the stock solutions were filtered using a 0.45 µm syringe filter. Control groups received medium without added plant material. The plates were then incubated.

At time point 48 h, after we added the experimental solutions to the HaCaT cells, the medium in all experimental groups and the untreated control group was aspirated and the cells were washed twice with 100 µL PBS. The dead cell control group was treated with Triton X-100 (0.2% in PBS) that was removed after 15 min. A volume of 180 µL serum and additive-free DMEM along with 20 µL MTT solution (5 mg/mL stock solution in PBS) was added to the experimental and control groups. The plates were then covered with aluminum foil and incubated for 90 min. After the incubation period, we added 200 µL DMSO to every experimental, control, and DMSO blank well to dissolve the formazan crystals that formed during incubation. The plates were consequently placed on an orbital shaker for 1 h to dissolve the formazan completely. After 1 h, the absorbance was measured at 560 nm and 630 nm.

Cell viability was consequently calculated using the following equation (Equation (1)):

% Cell viability = (ΔSample − ΔBlank)/(ΔControl − ΔBlank) × 100(1)

where

ΔSample = absorbance of treated cells_560_ – absorbance of treated cells_630_;
ΔBlank = mean absorbance of blank_560_ – mean absorbance of blank_630_;
ΔControl = mean absorbance of untreated control_560_ – mean absorbance of untreated control_630_.

### 2.6. In Vitro Wound Healing Scratch Assay

HaCaT cells were trypsinized at 80% confluence and counted with trypan blue using a hemocytometer (Marienfield-Superior, 0.0025 mm^2^, Paul Marienfeld GmbH & Co. KG, Lauda-Königshofen, Germany). A cell suspension was prepared, and cells were seeded at a density of 400,000 cells/mL. A volume of 2.5 mL of this prepared cell suspension was seeded into every well of a 12-well plate (Corning Costar Corporation, Corning, NY, USA). The plate was then incubated at 37 °C in a 5% CO_2_ humidified atmosphere for 24 h. After 24 h, the cells were visualized under a microscope to establish the formation of a monolayer in each well. Experimental solutions of the selected plant materials were prepared by preparing a stock solution of plant material in DMEM growth medium and diluting to appropriate concentrations (0.4, 0.6, and 1.3 mg/mL for *Aloe muth-muth* gel and whole leaf). Both *Aloe muth-muth* gel and whole leaf materials were tested for wound healing. Scratches were induced in the monolayers across the diameter of the wells using a sterile 200 µL pipet-tip [14,15]. The culture medium in each well was aspirated and each well was washed 4 times with serum- and additive-free DMEM. A volume of 4 mL of each concentration, as well as culturing medium only (untreated control), was added to the wells in triplicate. Photos of each well were taken immediately after scratches were induced and at 8 h intervals, thereafter, for a total period of 48 h. The photos were taken with a camera (The Imaging Source DFK 72AUC02) mounted on a Nikon TS100 light microscope (Nikon Instruments, Tokyo, Japan). For the duration of the experiments, we incubated the plates in a humidified environment with 5% CO_2_ at 37 °C. ImageJ software was used to measure the wound surface area, and the percentage wound closure was calculated according to the following modified equation [10]:

% Wound closure = [((Pre-migration)_surface area_ − (Migration)_surface area_)/(Pre-migration)_surface area_] × 100 (2)

where (Pre-migration)_surface area_ is the initial wound surface area (µm^2^) at time 0 h, and (Migration)_surface area_ is the wound surface area (µm^2^) at a specific time point. The closure rate (µm^2^/h) was calculated with the following modified equation [10]:

Closure rate (µm^2^/h) = [((Pre-migration)_surface area_ − (Migration)_surface area_)/Time (h)] (3)

### 2.7. In Vitro Cell Migration Assay

The effects of *Aloe muth-muth* gel (selected on the basis of the wound closure results) on cell migration was evaluated using the Cell Biolabs CytoSelect 24-Well Cell Migration Assay kit. The kit consisted of a 24-well cell culture plate with 12 polycarbonate membrane inserts (8 µm pore size). Individual cell suspensions containing 1 × 10^6^ cells/mL were prepared, having been resuspended in serum- and additive-free DMEM after sub-culturing. The suspensions were centrifuged at 140× *g* for 5 min. Each individual pellet was then resuspended in 1 mL serum- and additive-free DMEM containing the same working concentrations of *Aloe muth-muth* gel as used in the scratch assay (0.4, 0.6, and 1.3 mg/mL). The untreated control was resuspended in serum- and additive-free DMEM only. A volume of 500 µL of DMEM growth medium was added to each bottom well (outside the inserts). A volume of 300 µL of each prepared suspension containing experimental concentrations, as well as 300 µL of the untreated control, were added to the inside of the inserts in triplicate. The plates were then incubated in a humidified environment with 5% CO_2_ for 24 h. After the incubation period, we aspirated the media in each insert, and wetted cotton-tipped swabs were used to gently clean out the interior of each insert. Each insert was then transferred to a clean well containing 400 µL of the supplied staining solution and incubated for 10 min at room temperature. The inserts were gently rinsed using a beaker of water and allowed to air dry. Each insert was then added to another clean well containing 200 µL of the supplied extraction solution and incubated for 10 min on an orbital shaker. A volume of 100 µL of each sample was added to a 96-well microtiter plate and the absorbance was measured at 560 nm.

### 2.8. Statistical Analysis

All the experiments were performed in triplicate and the data were statistically analyzed with STATISTICA Version 12 (Statsoft, Tulsa, OK, USA). A one-way analysis of variance (ANOVA) followed by Tukey’s honest significant difference (HSD) test were performed, with statistically significant differences accepted at *p* < 0.05. The non-parametric Kruskal–Wallis test was used as verification. For the MTT assay results, we used the Bonferroni test to assess the statistically significant differences between each treatment and the untreated control (significance accepted at *p* < 0.05). The data were presented as mean (*n* = 3) ± standard deviation (error bars).

## 3. Results and Discussion

### 3.1. Characterization of Aloe Muth-Muth Whole Leaf and Gel Materials

**Figure 1:**
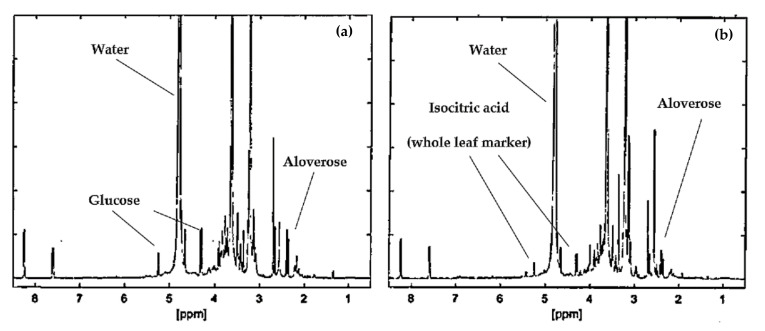
Quantitative proton nuclear magnetic resonance spectroscopy (^1^H-NMR) spectra for *Aloe muth-muth* gel (**a**) and *Aloe muth-muth* whole leaf (**b**) materials.

The ^1^H-NMR spectra for the gel and whole leaf materials are shown in Figure 1a,b, respectively. The content of marker molecules in the *Aloe muth-muth* gel and whole leaf materials obtained from quantitative ^1^H-NMR are shown in Table 1.

As expected, the composition as listed in Table 1 and Figure 1 for *Aloe muth-muth* is similar to that of *Aloe vera* and *Aloe ferox* with respect to the type and quantity of marker chemical compounds present [10].

### 3.2. Cytotoxicity Testing of Aloe Muth-Muth Gel and Whole Leaf Material Using the MTT Assay

**Figure 2:**
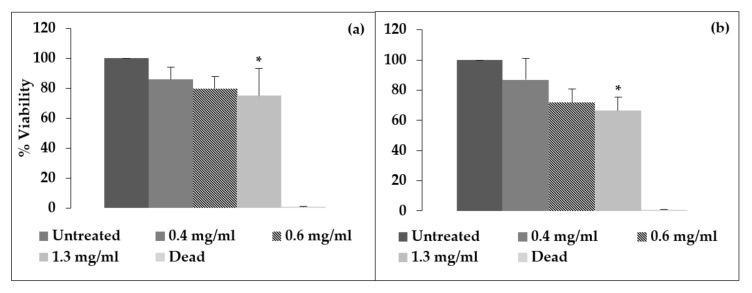
Percentage cell viability of immortalized human keratinocyte (HaCaT) cells after 48 h exposure to *Aloe muth-muth* gel (**a**) and *Aloe muth-muth* whole leaf (**b**) plant material. The data were normalized to the untreated control that is considered as 100% viable. The * indicates statistical significance in comparison to the untreated control (Bonferroni test, *p* < 0.05).

The MTT assay results for *Aloe muth-muth* gel and whole leaf materials after 48 h exposure are shown in Figure 2a,b, respectively. It is evident from the data depicted in Figure 2 that the *Aloe muth-muth* gel and whole leaf materials showed a concentration-dependent decrease in cell viability, as measured with the MTT assay after 48 h. At 24 h exposure, no significant effects were observed on the cell viability (results not shown). López-García et al. [16] provided guidelines on the cytotoxicity level of a compound on the basis of its effect on the in vitro cell viability percentage. A compound is considered non-cytotoxic when a cell viability of higher than 80% is obtained; weak cytotoxicity is associated with 60–80% cell viability, moderate cytotoxicity is linked with 40–60% viability, and a cell viability lower than 40% indicates strong cytotoxicity. Overall, both the *Aloe muth-muth* gel and whole leaf material displayed weak to non-cytotoxic properties and can therefore be considered safe to use on human skin. This is in line with previous findings for the gel and whole leaf materials of different aloe species [10,17].

### 3.3. Measuring the Re-Epithelialization Potential of Aloe Muth-Muth Gel and Whole Leaf Material Using the Scratch Assay

**Figure 3:**
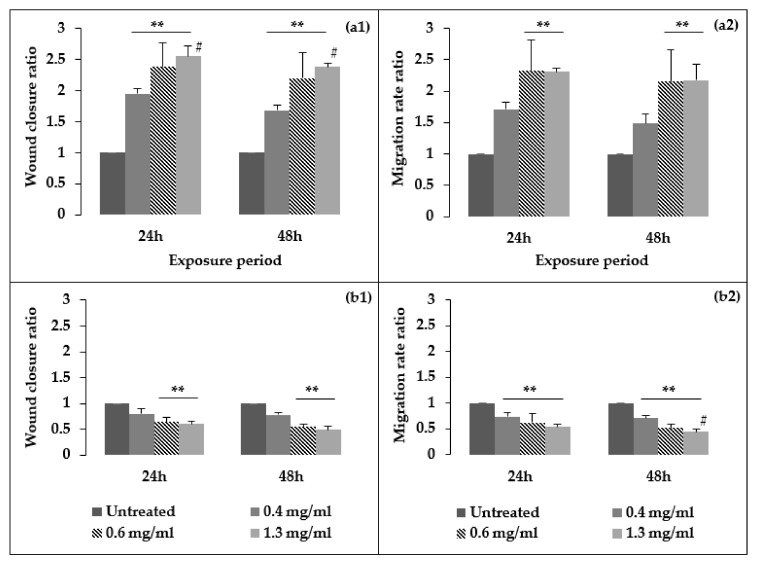
Ratio of percentage wound closure (1) and wound closure rate (2) results after exposure to (**a**) *Aloe muth-muth* gel and (**b**) *Aloe muth-muth* whole leaf at 24 h and 48 h treatment periods. Each data point was calculated with respect to the relevant initial wound value at time 0 h and normalized to the untreated control, which was considered as 1. The ** and # indicate statistical significance determined with Tukey’s honest significant difference (HSD) and Kruskal–Wallis tests, respectively, in comparison to the untreated control (significance accepted when *p* < 0.05).

**Figure 4:**
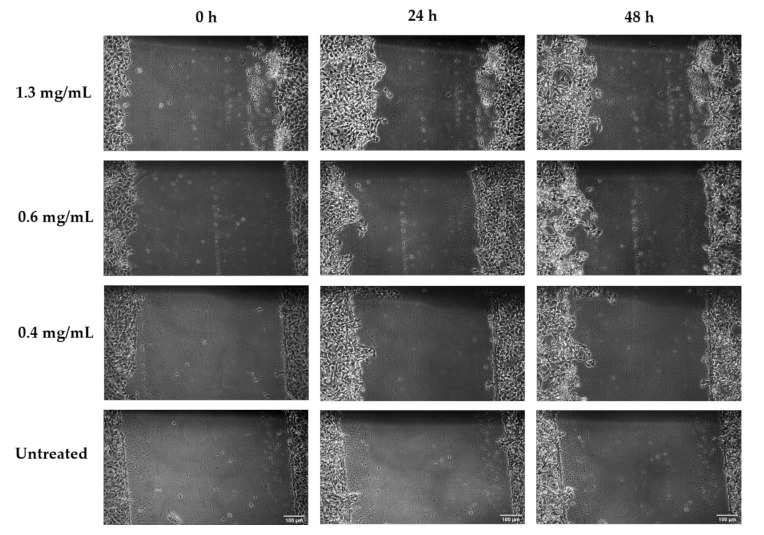
Microscopic photos of wound gaps in HaCaT cells introduced by the scratch technique after treatment with *Aloe muth-muth* gel at 1.3 mg/mL, 0.6 mg/mL, and 0.4 mg/mL, compared to an untreated control, at 0 h, 24 h, and 48 h. Images were captured at 10× magnification and the scale bars indicate 100 µm.

**Figure 5:**
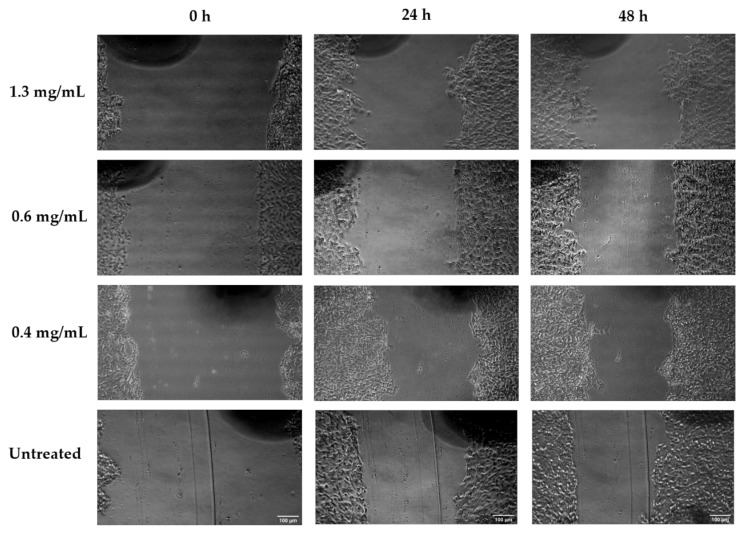
Microscopic photos of wound gaps in HaCaT cells introduced by the scratch technique after treatment with *Aloe muth-muth* whole leaf material at 1.3 mg/mL, 0.6 mg/mL, and 0.4 mg/mL, compared to an untreated control, at 0 h, 24 h, and 48 h. Images were captured at 10× magnification and the scale bars indicate 100 µm.

Figure 3 presents the percentage wound closure and migration rate results for *Aloe muth-muth* gel and whole leaf material as a ratio of the untreated control. Microscopic images depicting the wound gap closure results after 24 and 48 h of treatment with *Aloe muth-muth* gel and whole leaf material are depicted in Figure 4 and Figure 5, respectively. It is evident from Figure 4 that the application of *Aloe muth-muth* gel to HaCaT cells with scratched wound gaps resulted in notable improvement in wound gap closure in comparison to the untreated control. The wound closure results in Figure 3a1,a2 indicated the effectiveness of *Aloe muth-muth* gel with respect to wound healing, exhibiting 2-fold to 2.5-fold higher wound closure in comparison to the untreated control.

This notable improvement in wound closure, which was statistically significant (Tukey’s HSD test, *p* < 0.05), was obtained at all three concentrations of *Aloe muth-muth* gel investigated in this study. The improvement in wound healing for the *Aloe muth-muth* gel material was concentration-dependent, with the highest concentration of 1.3 mg/mL of the gel resulting in the highest percentage wound closure. In accordance with the percentage wound closure caused by the *Aloe muth-muth* gel material, we also found an increase in the closure rate (Figure 3a2) of the wounds in comparison to the untreated control, but only the wound closure rates of the 0.6 and 1.3 mg/mL concentrations were statistically significant (Tukey’s HSD test, *p* < 0.05). Several studies have reported *Aloe vera* gel to increase keratinocyte proliferation by upregulating the expression of vascular endothelial growth factor-A (VEGF-A) [18,19]. The major compounds responsible for this phenomenon are aloesin, aloin, and emodin. The gel and whole leaf extracts of different *Aloe* species have also shown the ability to hydrate the skin and reduce erythema, which can be attributed to the polysaccharides such as aloverose that are present in both the gel and whole leaf [20].

In contrast to the promising wound healing results obtained with the *Aloe muth-muth* gel, the *Aloe muth-muth* whole leaf material (Figure 3b1,b2 and Figure 5) did not give similar wound healing results. Both wound gap closure and closure rate were not increased in comparison to the untreated control. This can possibly be attributed to differences in the chemical composition of the whole leaf material compared to the gel material. As shown in Table 1, the whole leaf material contained less aloverose (a bioactive polysaccharide in aloe gel material) than the gel material, but more citric acid and iso-citric acid. These chemical differences were due to the inclusion of leaf rind material in the *Aloe muth-muth* whole leaf material that was not part of the *Aloe muth-muth* gel material. Citric acids have been shown to induce cell-cycle arrest and apoptosis via the caspase- and mitochondrial-dependent signaling pathways in HaCaT cells [21].

### 3.4. Measuring the Migration Enhancement Activity of Aloe muth-muth Gel Using the In Vitro Cell Migration Assay

In general, the lowest concentration (0.4 mg/mL) of the *Aloe muth-muth* gel resulted in a decrease in cell migration compared to the untreated control, while a concentration-dependent improvement in cell migration was observed for the 0.6 and 1.3 mg/mL concentrations (results not shown). The increase in cell migration in comparison to the untreated control was, however, not statistically significant (*p* > 0.05). Therefore, at higher *Aloe muth-muth* gel concentrations, cell migration may play a possible role in the wound healing effect. Aloesin is a compound in *Aloe* species that has been shown to increase cell migration via the phosphorylation of cytokines and growth factors [22].

## 4. Conclusions

*Aloe muth-muth* gel and whole leaf materials were investigated for wound healing properties using the scratch assay with the HaCaT cell culture model. MTT assays indicated that none of the plant materials at the concentration ranges used in this study showed cytotoxic effects against HaCaT cells. *Aloe muth-muth* gel exhibited significant wound healing properties as indicated by a statistically significant increase in the percentage wound gap closure and migration rate for the two highest concentrations used in this study, in comparison to an untreated control. *Aloe muth-muth* whole leaf material showed some wound healing effects, but to a much lower extent than the gel material, and this was not statistically significant. In conclusion, gel and whole leaf extract of the newly cultivated species, *Aloe muth-muth,* showed potential as a wound healing therapeutic agent in future drug development strategies, following in the footsteps of the species from which it was cultivated, namely, *Aloe vera* and *Aloe ferox*.

## Figures and Tables

**Table 1:** Quantity of marker molecules in *Aloe muth-muth* gel and whole leaf materials determined by ^1^H-NMR spectroscopy.

Component	*Aloe muth-muth* Gel		*Aloe muth-muth* Whole Leaf	
	Content (%)	Content (mg/mL)	Content (%)	Content (mg/mL)
Aloverose (polysaccharide)	11.3	793.8	8.1	568.3
Glucose	11.7	821.1	6.8	477.2
Malic acid	10.4	730.8	5.4	380.3
Lactic acid	0.1	12.5	Traces	ND
Citric acid	ND	ND	1.5	103.8
Iso-citric acid (whole leaf marker)	ND	ND	5.1	355.8

Key to abbreviations: ND—not detected.
